# The Lettsomian Lectures

**Published:** 1895-02-02

**Authors:** 


### The Lettsomian Lectures.

Dr. Roberts has done good service in drawing attention to the combinations of morbid conditions which go to make up the maladies from which patients suffer. He takes as his text those mixed conditions which are found within the chest, but the same principle is of far wider application. As aiding comparison and as encouraging exactitude, nosology is an important branch of medicine. Life is too short for us to describe every case, we must have words to express commonly repeated morbid states, and thus we give names to diseases. But one of the first teachings of experience is that the names and the maladies will not always fit, and that, as Dr. Roberts tells us, the cases we see in practice are not "cut and dried examples of individual diseases," as they are described in text-books. One result of this has been the multiplication of so-called diseases and their subdivision to such an extent that the mere finding of names for them has become a difficulty. Mr. Hutchinson, in despair of finding for strange and rare conditions names which shall mark a difference and still not convey false pathological ideas, has even gone so far as to ticket these conditions provisionally with the names of the patients in the history of whose cases they have been first described; so that, in advance of our defined nosologies, we have the growing points of medical science docketted as Smith's, or Jones', or Robinson's diseases.

It is clear, however, that many of the cases which are unconformable and tally ill with classical descriptions are but examples of what Dr. Roberts describes as "combinations of morbid conditions." Among the instances which he gives as illustrating the complicated conditions which may together go to the making up of the "case," as it is presented to us for treatment and prognosis, he cites that of chronic phthisis. Here the tendency is to concentrate attention too much upon bacilli and not to appreciate sufficiently the highly complicated state towards which the lungs and air passages ultimately tend. There may be distinct masses of consolidation, partly tuberculous, partly inflammatory, partly caseous; miliary tubercles, diffused or in groups; softening of these structures or breaking down of the lung itself; cavities of all shapes and sizes, with suppuration taking place within them; processes of repair, with development of fibroid tissue and contraction and induration of the lung. Associated with these there may be bronchitis, causing expectoration, which is not tuberculous, or there may also be definite tuberculous ulcers in the larger bronchi or the trachea, causing tuberculous expectoration not from the lung. Then in parts not actually phthisical, compensatory distension, or emphysema, may be met with, or aneurisms may develop on branches of the pulmonary artery. In addition to all these there may be every variety of pleurisy, from a mere thickening to a pyo-pneumo-thorax. All these may be jumbled together, and unless we bear in mind that phthisis is not a process but a score of processes, the confusion will be meaningless and inextricable. This is but an example; in diseases of the heart, or bronchi, or pleura it is the same, as, indeed, is the case in every malady. Measles with bronchitis and measles without are prognostically two diseases; an accident or operation in a patient with good kidneys differs entirely from the same affection when the kidneys are incompetent. Many tunes can be played on a few strings, the character of a note depends on the chord of which it is a part; and so in medicine the nosology of the text book is useful for the student, individual diseases must first be understood, but the thing which the practitioner has to study and to treat is not the "cut and dried example," but the particular mixture of diseased conditions which the case presents.

